# Stochastic Resonance in a Small-World Network of Rulkov Neurons with Electrical and Chemical Synapses Under an Alternating Electric Field and Synaptic Transmission Delay

**DOI:** 10.3390/biomimetics11100733

**Published:** 2026-10-14

**Authors:** Chunhua Yuan, Yunrong Jiang, Junfeng Wang, Xiangyu Li

**Affiliations:** School of Automation and Electrical Engineering, Shenyang Ligong University, Shenyang 110159, China; yuanch@sylu.edu.cn (C.Y.); 2506620404@stu.sylu.edu.cn (Y.J.); 13470200991@163.com (J.W.)

**Keywords:** stochastic resonance, alternating electric field, small-world network, electrical and chemical synapses, synaptic transmission delay

## Abstract

Biological neural systems can use moderate noise to enhance their responses to weak stimuli, and this process is affected by synaptic transmission, network structure, transmission timing, and external electric fields. We constructed a Watts–Strogatz network of Rulkov neurons with electrical and chemical synapses, Gaussian noise, an alternating electric field, and synaptic transmission delay to examine stochastic resonance in response to a periodic signal that produced a weak noise-free population response at its fundamental frequency. All tested network configurations showed a maximum response at an intermediate noise intensity. Increasing the chemical-synapse probability increased the peak response in the initial synaptic-composition scan, whereas the effect of network rewiring varied with synaptic composition. After transmission delay was introduced, the field setting associated with a stronger response varied with synaptic composition and delay. Several delay-dependent response maxima shifted when the input period changed. These results characterize how synaptic composition, transmission delay, and external-field settings shaped the input-frequency response within the modeled neuronal network.

## 1. Introduction

Noise is an intrinsic component of neuronal activity. In nonlinear excitable systems, an intermediate noise intensity can maximize the response to a weak periodic input, a phenomenon known as stochastic resonance (SR) [1,2]. Experimental studies of sensory neurons have shown that SR can improve the detection and transmission of subthreshold signals [3]. When noise intensity is matched to neuronal excitability and the external stimulus, a weak signal that would otherwise fail to produce a reliable response may become detectable. This property has motivated bio-inspired strategies for processing weak periodic signals in noisy environments.

At the network level, noise-assisted responses to weak signals are affected by both network topology and synaptic transmission. Watts–Strogatz networks combine relatively high clustering coefficients with short characteristic path lengths and provide a compact representation of the small-world network observed in many complex systems, including brain networks [4,5]. Signal transmission is also affected by the combined action of electrical and chemical synapses. Electrical synapses typically provide direct coupling through membrane-potential differences, whereas chemical synapses transmit signals through presynaptic-to-postsynaptic interactions [6]. Small-world topology describes network connectivity, whereas a hybrid synaptic architecture combines electrical and chemical transmission. Together, these features can influence signal-propagation pathways and the conditions under which neurons are activated.

Simulations of a hybrid scale-free network of FitzHugh–Nagumo neurons showed that synaptic composition and coupling strength affected SR and that electrical synapses produced a stronger input–output correlation than chemical synapses under the tested conditions [7]. In hybrid-coupled small-world neuronal networks, the probability of assigning chemical synapses to network edges, the coupling strengths, and the rewiring probability can alter SR and the propagation of localized subthreshold inputs [8]. These effects also depend on synaptic transmission delay and on whether electrical and chemical coupling are combined or examined separately [9,10,11]. Studies of inhibitory and other coupling configurations further indicate that the contributions of the two synapse types depend on whether chemical coupling is excitatory or inhibitory, as well as on the coupling architecture and neuronal dynamics [12,13,14,15]. The same change in network topology may therefore produce different responses under different synaptic compositions. This raises the question of whether the effect of small-world rewiring on SR depends on the balance between electrical and chemical synapses.

Time-varying electric fields provide an external means of regulating neuronal excitability and population activity. Experimental studies have shown that alternating-current stimulation can alter cortical network activity and phase locking [16,17]. Computational studies have examined the effects of induced electric fields and electromagnetic induction on neuronal firing and resonance [18,19,20,21,22,23,24,25,26]. In a Hodgkin–Huxley neuronal network, magnetic-field parameters altered stochastic resonance under Gaussian and Lévy channel noise; resonance amplitude varied with magnetic-field strength and was not consistently greater at stronger fields [27]. Liu et al. examined envelope-modulated signals in networks with different topologies under electromagnetic induction and Gaussian white noise. They found that the noise intensity associated with resonance and the effective range of electrical coupling varied across topologies [28]. Related work has also examined resonance under electromagnetic induction in multilayer networks [29]. Simulations of a two-compartment neuron showed that the effects of an AC-induced electric field varied with field amplitude and frequency [30]. In an olive-neuron model, subthreshold resonance depended on stimulus intensity and on the timescales of the fast and slow dynamics [31]. These findings motivate a comparison of field-related responses across synaptic compositions and an examination of how transmission delay modifies those responses.

Synaptic transmission delay represents the finite time required for a signal to pass from a presynaptic neuron to a postsynaptic neuron and introduces an internal timescale that differs from the period of the external stimulus. Because synaptic input arrives after transmission, delay changes its timing relative to the external periodic signal. Previous studies of neuronal networks have shown that delay can enhance or suppress SR and can produce multiple response maxima at selected delays [9,10,21,32,33,34]. In excitable FitzHugh–Nagumo neuronal networks, Yu et al. found that bounded cross-correlated noise and time delay affected subthreshold signal transmission; tuning the delay produced resonance peaks at half-integer multiples of the signal period [32]. Wu and Sun reported delay-induced stochastic multiresonance in small-world FitzHugh–Nagumo networks. Under moderate noise, several response maxima occurred near integer multiples of the subthreshold signal period, and their strength varied with network parameters [35]. In other delayed coupled systems, network topology, noise type, and coupling structure can also change the distribution of delay-dependent resonance maxima [36,37]. Delayed electrical and chemical coupling can produce different synchronization transitions [12,13]. More recent work has examined delay-dependent responses in other network architectures, including multilayer networks under electromagnetic induction [29,38,39]. These findings show that delay-dependent responses vary with noise, network structure, and model conditions. We therefore vary the input period to test how delay-dependent maxima shift with the external timescale.

Previous studies have examined hybrid synapses, small-world topology, electric fields, and transmission delay in relation to SR. Here, we examine their combined effects in one network by comparing how synaptic composition conditions the response to rewiring, how the field setting associated with a stronger response varies across synaptic compositions, and how transmission delay modifies field-dependent responses. We also vary the input period to characterize shifts in delay-dependent response maxima.

To address these questions, we constructed a two-dimensional Rulkov-neuron network with Watts–Strogatz connectivity, electrical and chemical synapses, independent Gaussian noise, an alternating electric field, and synaptic transmission delay [40,41]. We compare noise-dependent responses across synaptic compositions and coupling strengths, examine how synaptic composition affects the response to rewiring and electric-field forcing, and vary transmission delay and input period to characterize the relation between synaptic-input timing and the external periodic signal. The analysis focuses on response patterns across the parameter ranges examined in this network model.

## 2. Model and Methods

The neuronal network consists of N=200 two-dimensional Rulkov neurons connected according to the Watts–Strogatz small-world model. Initially, the neurons are arranged in a regular ring lattice. For each node *i*, edges are added to nodes i+1,…,i+K, with indices wrapped around the ring; thus, each node has 2K=6 neighbors when K=3. Each original ring edge (i,j) is considered once in the prescribed loop order and is rewired with probability *p*. When selected, the edge is removed, and node *i* is connected to a node selected uniformly from those that are neither *i* nor its current neighbors. This procedure excludes self-connections and duplicate edges [4]. Unless otherwise specified, the rewiring probability is p=0.1.

After rewiring, let *M* denote the number of undirected edges. For a chemical-synapse probability $p_{t}$, $m=\mathrm{round}(Mp_{t})$ edges are sampled uniformly without replacement. For each selected edge, one of its two directed entries is chosen at random and removed, leaving exactly one directed chemical connection. At $p_{t}=1$, every underlying undirected edge is represented by one directed chemical connection. Edges not selected remain bidirectional and are assigned as electrical synapses. Network topology, chemical-synapse assignments, and directions are generated or reused across scan points according to the scan-specific protocol summarized in Supplementary Table S2.

The dynamics of each neuron are described by the two-dimensional Rulkov map in Equation (1) [40,41]. The map provides a computationally efficient representation of neuronal spiking and bursting dynamics for network-level simulations:

(1) $$\begin{array}{cc} x_{i}\left(n+1\right) & =\frac{\alpha }{1+{\left[x_{i}\left(n\right)+V_{e}\right]}^{2}}+y_{i}\left(n\right)+I_{i}^{\mathrm{syn}}\left(n\right)+\xi_{i}\left(n\right)+I_{e},\\ y_{i}\left(n+1\right) & =y_{i}\left(n\right)-\beta x_{i}\left(n\right)-\gamma . \end{array}$$

The fast dynamical variable $x_{i}$ represents the membrane potential, whereas the slow recovery variable $y_{i}$ represents the recovery process. The terms $I_{i}^{\mathrm{syn}}$, $\xi_{i}$, $V_{e}$, and $I_{e}$ denote the total synaptic current, additive noise, field-induced voltage, and associated capacitive current, respectively. A weak periodic input was applied as a square-pulse train. Shared neuronal and input parameters are listed in Table 1.

The total synaptic current is $I_{i}^{\mathrm{syn}}=I_{i,e}^{\mathrm{syn}}+I_{i,c}^{\mathrm{syn}}$. Bidirectional edges represent electrical synapses and continuously couple differences in the fast variable. Their contribution is defined by Equation (2):

(2) $$I_{i,e}^{\mathrm{syn}}\left(n\right)=g_{e}\sum_{\begin{array}{c} j=1\\ j\neq i \end{array}}^{N}C_{e}\left(i,j\right)\left[x_{j}\left(n-\tau_{e}\right)-x_{i}\left(n\right)\right].$$

Here, $C_{e}$ is the electrical connectivity matrix, $g_{e}$ is the electrical coupling strength, and $\tau_{e}$ is the electrical-synapse transmission delay. In Equation (2), the delay is applied to the presynaptic state $x_{j}\left(n-\tau_{e}\right)$; the postsynaptic state remains $x_{i}\left(n\right)$. Chemical synapses are unidirectional and use threshold-dependent transmission [8,9,11]. Their contribution is defined by Equation (3):

(3) $$I_{i,c}^{\mathrm{syn}}\left(n\right)=g_{c}\left[v_{i}-x_{i}\left(n\right)\right]\sum_{\begin{array}{c} j=1\\ j\neq i \end{array}}^{N}C_{c}\left(i,j\right)\Gamma \;\left(x_{j}\left(n\right)\right).$$

The presynaptic gating function is given by Equation (4):

(4) $$\Gamma \;\left(x_{j}\left(n\right)\right)=\frac{1}{1+\exp\left\{-\lambda \left[x_{j}\left(n-\tau_{c}\right)-\Theta_{s}\right]\right\}}.$$

In Equations (3) and (4), $C_{c}$ is the chemical connectivity matrix, $g_{c}$ is the chemical coupling strength, and $\tau_{c}$ is the chemical-synapse transmission delay. Equation (4) defines the sigmoid gating function for presynaptic activity; $\Theta_{s}$ and λ are its center and steepness parameter, respectively, with values listed in Table 1. The reversal potential $v_{i}$ depends on the chemical-input assignment of each node, as specified in the same table. The synaptic current enters the fast-variable equation with the driving term shown in Equation (3). In the figures, $D_{e}$ and $D_{c}$ denote $g_{e}$ and $g_{c}$, respectively. The parameter $p_{t}$ denotes the probability that an edge is assigned a chemical synapse, hereafter termed the chemical-synapse probability.

The periodic forcing is provided by an alternating electric field, which enters the fast-variable equation through $V_{e}$ and $I_{e}$. The field-induced voltage satisfies $V_{e}=\Delta V$, as defined by Equation (5):

(5) $$\Delta V=-\frac{A}{\omega_{e}}\sin\left(\omega_{e}n\right).$$

The associated capacitive current is defined by Equation (6):

(6) $$I_{e}=A\cos\left(\omega_{e}n\right).$$

The amplitude *A* sets the perturbation magnitude, and $\omega_{e}$ denotes the angular frequency. Numerical scans use E≡A and $f=\omega_{e}/\left(2\pi \right)$. Recovery, synaptic input, and noise may alter the phase distribution of threshold crossings under this forcing [18,19,20,21,22,23,24,25,26,29].

All delay scans use $\tau_{e}=\tau_{c}=\tau$. This common effective delay provides a controlled comparison of synaptic-input timing across coupling configurations. Electrical and chemical synapses have distinct transmission dynamics; the shared τ is an effective timing parameter for the present comparison, while the two pathways retain their distinct coupling rules [12,13]. Electrical and chemical delays shift synaptic input relative to the weak pulse. A response maximum may occur when delayed synaptic input arrives during a favorable phase of the stimulus [9,10,21,32,33,34,36,37]. The observed maximum can reflect the combined influence of pulse timing, recovery dynamics, network propagation, and the resolution of the delay scan.

The response of the neuronal population at the input frequency is quantified by the Fourier response amplitude *Q* in Equation (7):

(7) $$\begin{array}{cc} \bar{x}\left(n\right) & =\frac{1}{N}\sum_{i=1}^{N}x_{i}\left(n\right),\\ Q_{\sin} & =\frac{2}{L}\sum_{n=n_{0}}^{n_{1}}\bar{x}\left(n\right)\sin\left(\omega_{s}n\right),\\ Q_{\cos} & =\frac{2}{L}\sum_{n=n_{0}}^{n_{1}}\bar{x}\left(n\right)\cos\left(\omega_{s}n\right),\\ Q & =\sqrt{Q_{\sin}^{2}+Q_{\cos}^{2}}. \end{array}$$

Here, $L=n_{1}-n_{0}+1$, $\omega_{s}=2\pi /T_{s}$, and $\bar{x}\left(n\right)$ denotes the average membrane potential of the neuronal population. The analysis intervals, including the starting index, record length, and relation between *L* and $T_{s}$, are specified by figure in Supplementary Table S2. A larger *Q* indicates a stronger population response component at the input fundamental frequency [1,2,8,9].

For the period–delay analysis, spectral SNR was calculated after subtracting the mean from the evaluated population signal. Signal power was obtained from the Fourier bin indexed by $\mathrm{round}(L/T_{s})+1$, where *L* is the evaluated record length. Background power was calculated as the mean power of the ten adjacent bins on each side of the signal bin, with the signal bin excluded. SNR was expressed as $10\log_{10}$ of the signal-to-background power ratio.

At each node and iteration, the additive noise term was generated as $\xi_{i}\left(n\right)=\sigma \eta_{i}\left(n\right)$, where the samples $\eta_{i}\left(n\right)$ were drawn independently from the standard normal distribution, N(0,1). Each dynamical curve was estimated from 20 simulation replicates. The curves show replicate-averaged values and two-sided 95% confidence intervals calculated using Equation (8):

(8) $$\bar{Q}\;\pm \;t_{0.975,n_{r}-1}\frac{s_{Q}}{\sqrt{n_{r}}},$$

where $n_{r}$ is the number of simulation replicates and $s_{Q}$ is the sample standard deviation of the replicate-level responses. All dynamical response curves use 20 replicates and the two-sided 95% Student-*t* critical value $t_{0.975,19}=2.0930$. Figure-specific scan conditions are stated in the corresponding Results descriptions and captions.

Each replicate was a complete model run under the scan-specific protocol in Supplementary Table S2, which specifies how network topology, chemical-synapse assignments and directions, and Gaussian noise were resampled or reused across conditions; reused realizations were paired. For the input-period delay scans, each replicate used a newly generated network and noise realization at each $T_{s}$, and these realizations were held fixed across all delays within that replicate. The topology-only analysis used 100 networks per rewiring probability and excluded dynamical noise.

## 3. Results

We first examined how synaptic composition, coupling strength, and network rewiring shaped the noise-dependent response. These analyses test how the modeled diversity of synaptic transmission and small-world network affect weak-signal enhancement. We then compared electric-field effects before and after introducing transmission delay. The final analysis varied the input period to examine the timing of the delay-dependent maxima. Throughout, the height of a *Q* peak describes the response at the input frequency, while its position along the noise or delay axis identifies the corresponding operating condition.

### 3.1. Effect of Synaptic Composition on Stochastic Resonance

**Figure 1:**
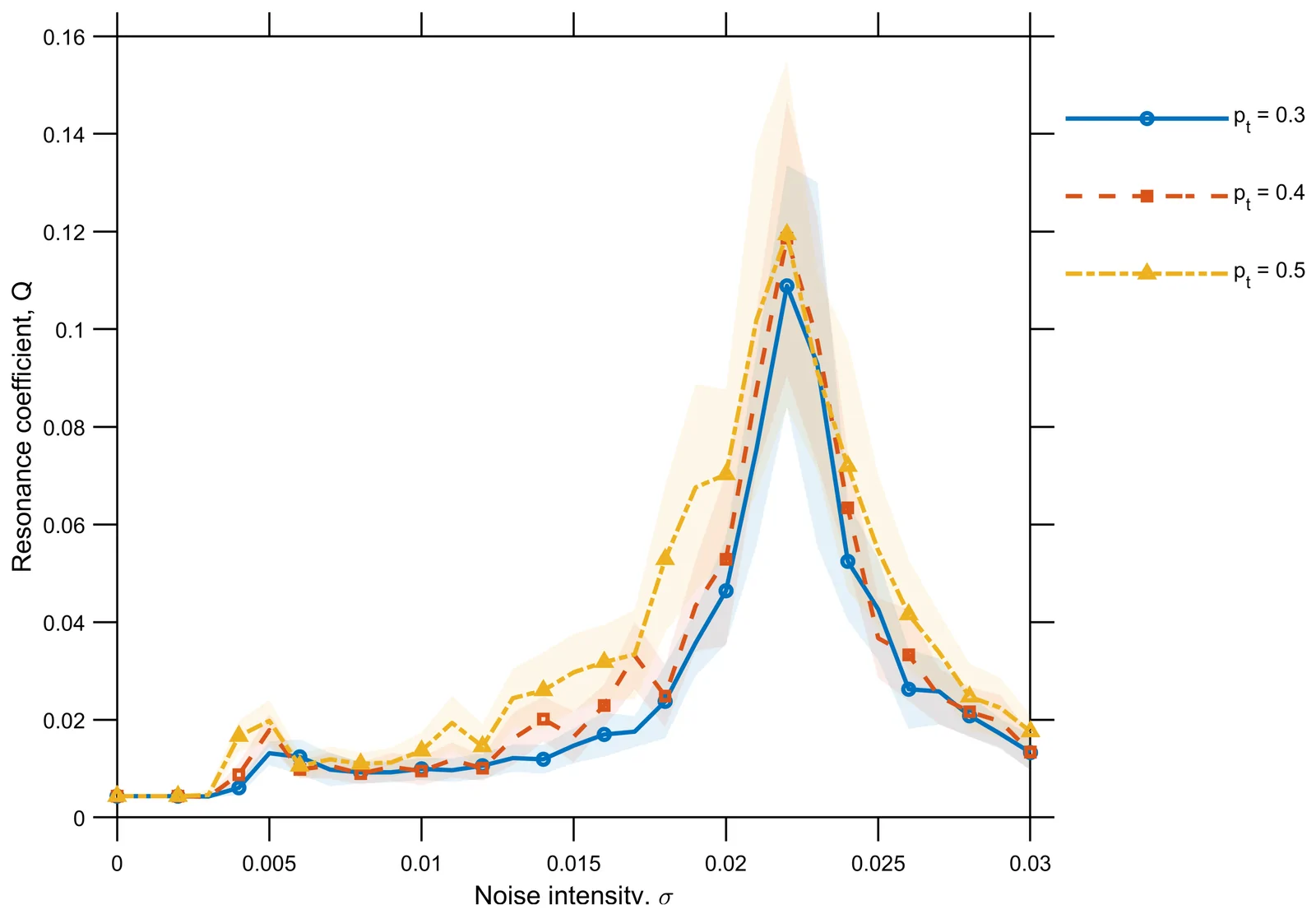
Stochastic-resonance response across synaptic compositions. Fourier response amplitude *Q* is plotted against noise intensity σ=0:0.001:0.030 for chemical-synapse probabilities $p_{t}=0.3$, 0.4, and 0.5 under zero-delay conditions, with N=200, K=3, p=0.1, α=1.95, β=γ=0.001, $D_{e}=0.003$, $D_{c}=0.010$, E=0.005, f=0.05, and $T_{s}=700$. The pulse was applied to node 1. Lines and shaded bands denote the mean and two-sided 95% Student-*t* confidence interval.

The chemical-synapse probability $p_{t}$ changes the mixture of electrical and chemical connections while retaining the underlying undirected edge set. We compared three compositions under zero-delay conditions to examine whether this change affected the magnitude or noise location of the SR response (Figure 1). All three curves rose as noise intensity increased, reached a maximum at an intermediate value, and then declined. The shared bell-shaped profile shows that the response remained noise dependent across the examined compositions.

The three maxima occurred at the same sampled noise intensity, σ=0.022. Peak *Q* was 0.1089 at $p_{t}=0.3$, 0.1187 at $p_{t}=0.4$, and 0.1194 at $p_{t}=0.5$. The peak mean therefore increased by 9.7% between $p_{t}=0.3$ and 0.5. The two-sided 95% Student-*t* confidence-interval half-widths were 0.0247, 0.0282, and 0.0356, respectively. The intervals overlapped, so we report the 9.7% increase as a descriptive difference between mean peak responses. Additional peak values are listed in Supplementary Table S1.

We quantified the population response in the absence of noise by setting σ=0, keeping the other Figure 1 settings unchanged, and performing 20 realizations for each $p_{t}$. The mean response at the input fundamental frequency was 0.004343, 0.004343, and 0.004342 for $p_{t}=0.3$, 0.4, and 0.5, respectively. These values were 3.6–4.0% of the corresponding noise-dependent peak responses at σ=0.022. In this manuscript, “subthreshold” describes this weak noise-free population response at the input fundamental frequency. For the reference setting E=0.005 and f=0.05, the model gives an $I_{e}$ amplitude of 0.005 and a $V_{e}$ amplitude of approximately 0.0159, compared with the square-pulse amplitude $A_{s}=0.0015$. The reported enhancement therefore refers to the increase in the population response at the input frequency under noise, relative to its zero-noise response in this field-driven model.

### 3.2. Effects of Electrical and Chemical Coupling Strengths on Stochastic Resonance

**Figure 2:**
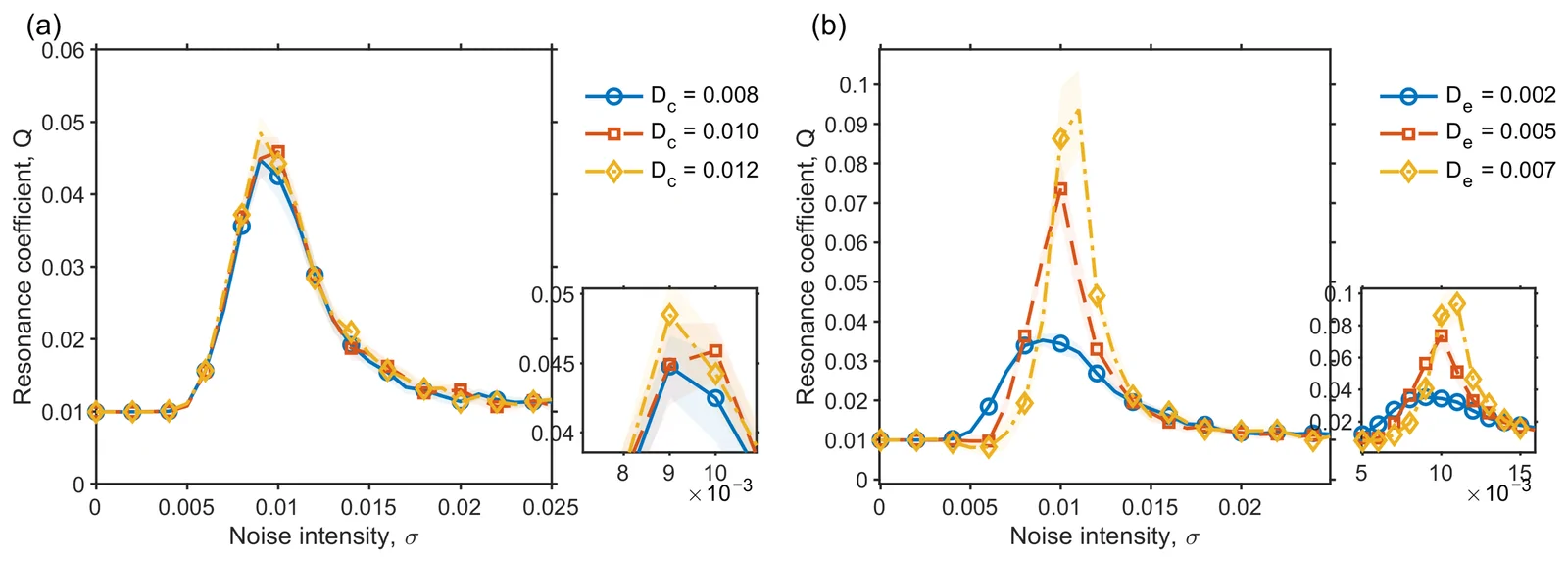
Stochastic-resonance responses to chemical and electrical coupling strength. Fourier response amplitude *Q* is plotted against noise intensity σ under global periodic stimulation for (**a**) $D_{c}=0.008$, 0.010, and 0.012 at fixed $D_{e}=0.003$, and (**b**) $D_{e}=0.002$, 0.005, and 0.007 at fixed $D_{c}=0.010$. Here, N=200, K=3, p=0.1, $p_{t}=0.2$, E=0.005, and $T_{s}=700$. Insets enlarge the peak region; lines and bands show means and two-sided 95% Student-*t* confidence intervals over 20 replicates.

Changing $p_{t}$ alters which connections carry chemical or electrical signals; changing $D_{c}$ or $D_{e}$ instead varies the strength of an existing coupling pathway. We scanned the two strengths separately under network-wide periodic stimulation to compare how each affected the SR curve (Figure 2). In Figure 2a, increasing $D_{c}$ from 0.008 to 0.012 slightly raised peak *Q*, from 0.0448 to 0.0485. The three curves remained closely grouped around σ=0.009–0.010, indicating a modest change in peak magnitude across the chemical-coupling interval.

The electrical-coupling scan separated the curves more clearly (Figure 2b). With $D_{c}=0.010$, peak *Q* rose from 0.0352 at $D_{e}=0.002$ to 0.0937 at $D_{e}=0.007$, while the peak location moved from σ=0.009 to 0.011. Across the respective scanned intervals, the change in $D_{e}$ therefore produced a larger variation in response amplitude than the change in $D_{c}$. The intermediate peak values are reported in Supplementary Table S1.

### 3.3. Effect of Network Rewiring on Stochastic Resonance Across Synaptic Compositions

**Figure 3:**
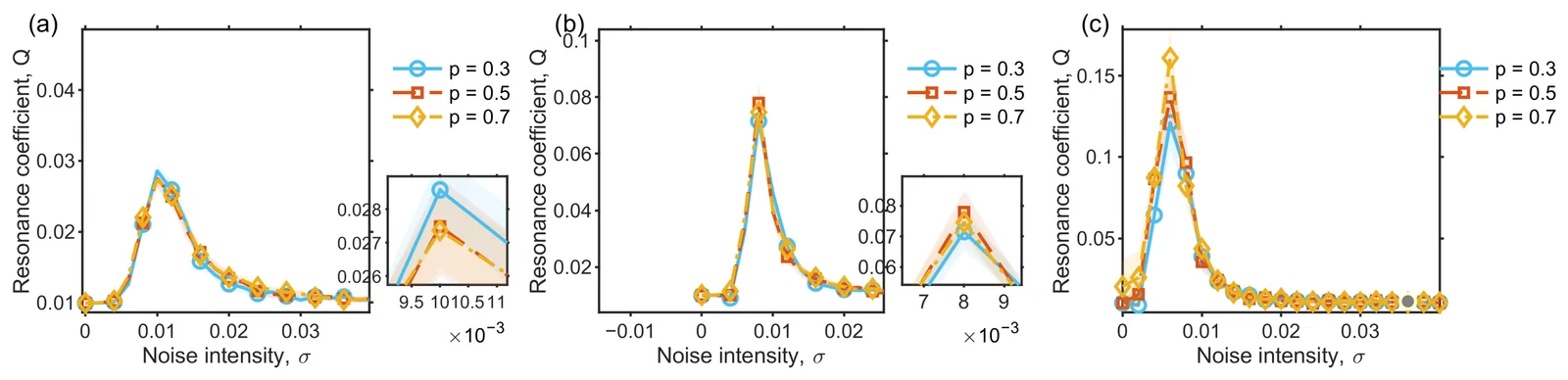
Synaptic-composition-dependent response to network rewiring. Fourier response amplitude *Q* is plotted against noise intensity σ for rewiring probabilities p=0.3, 0.5, and 0.7 at (**a**) $p_{t}=0.1$, (**b**) $p_{t}=0.3$, and (**c**) $p_{t}=0.5$, with $D_{e}=0.003$, $D_{c}=0.010$, E=0.005, f=0.05, and zero delay. The periodic square-pulse input was applied to node 1. Lines and bands show means and two-sided 95% Student-*t* confidence intervals over 20 replicates.

Network rewiring changes the paths available for network activity. To examine whether its effect depended on synaptic composition, we varied *p* from 0.3 to 0.7 at $p_{t}=0.1$, 0.3, and 0.5 (Figure 3). Every curve retained an intermediate-noise maximum, but the ordering of the peak heights across rewiring probabilities changed with $p_{t}$. Thus, the response to the same topological perturbation depended on the synaptic configuration.

At $p_{t}=0.1$, increasing *p* from 0.3 to 0.7 slightly reduced the peak, from 0.0286 to 0.0274. At $p_{t}=0.3$, the largest peak occurred at the intermediate rewiring probability, p=0.5. The clearest increase appeared at $p_{t}=0.5$, where peak *Q* rose from 0.1210 to 0.1610 across the same rewiring interval. The three response profiles therefore differed in direction and shape across the examined compositions. Supplementary Table S1 contains the reported peak series.

**Figure 4:**
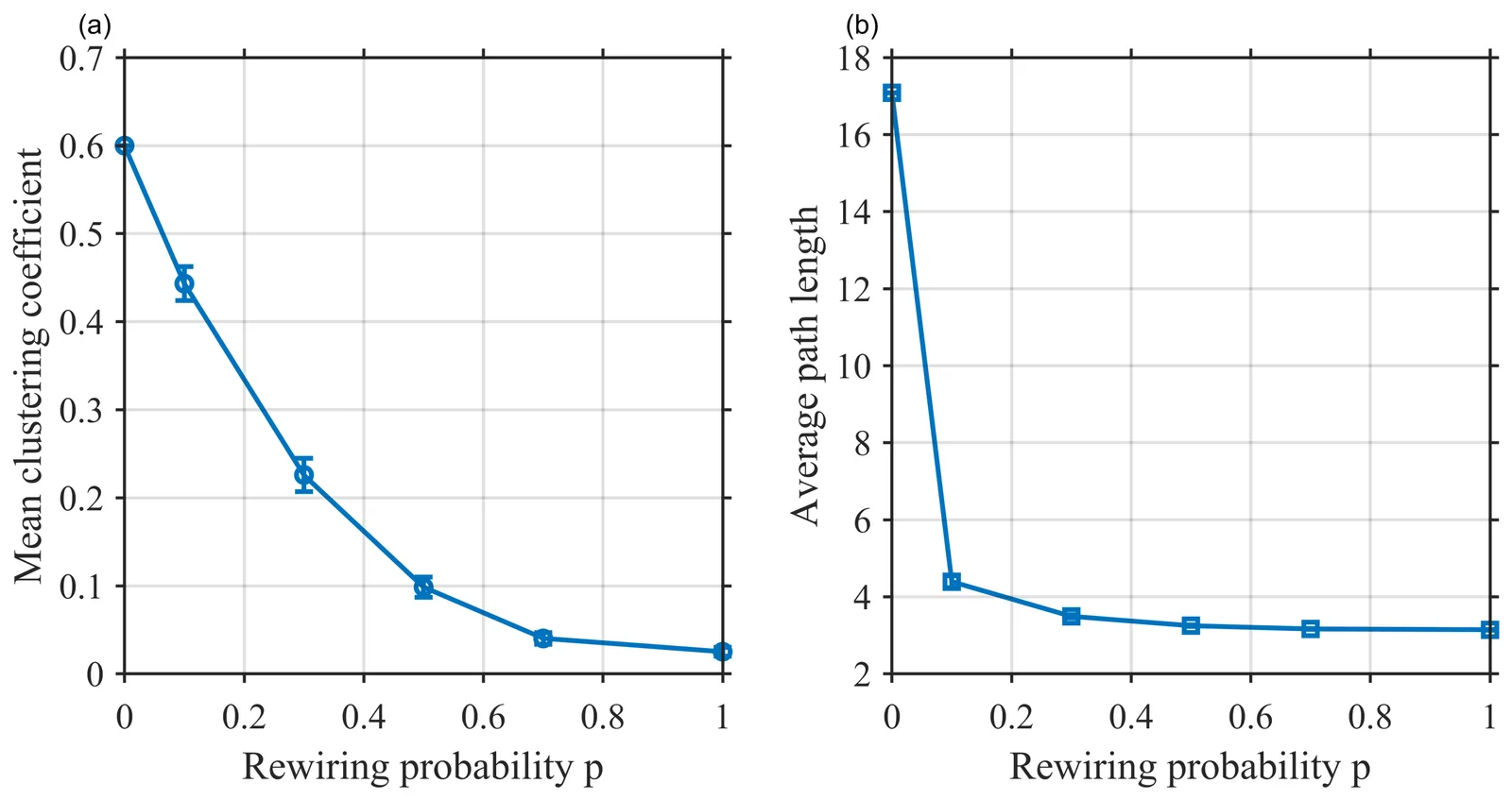
Changes in Watts–Strogatz network metrics with rewiring probability. (**a**) Mean clustering coefficient and (**b**) average shortest path length are shown as functions of *p* for networks with N=200 and K=3. Error bars denote standard deviations over 100 independently generated networks.

The topology measurements place this response in the context of the network changes induced by rewiring (Figure 4 and Table 2). Across 100 realizations, increasing *p* from 0 to 1 reduced both mean clustering coefficient and average shortest path length while preserving a mean degree of six. At the default p=0.1, path length had already fallen from 17.0854 to 4.3930, while clustering remained at 0.4433 compared with 0.6000 in the ring network. The configuration therefore combined short paths with substantial local clustering.

Within the narrower interval used for the SR scans, path length decreased from 3.4937 to 3.1670 and clustering from 0.2260 to 0.0403. The response profiles in Figure 3 therefore arose as these two network properties changed together. Their differing peak patterns across $p_{t}$ indicate that synaptic composition conditioned the response to this combined topological change.

### 3.4. Effects of Alternating Electric-Field Amplitude and Frequency on Stochastic Resonance

**Figure 5:**
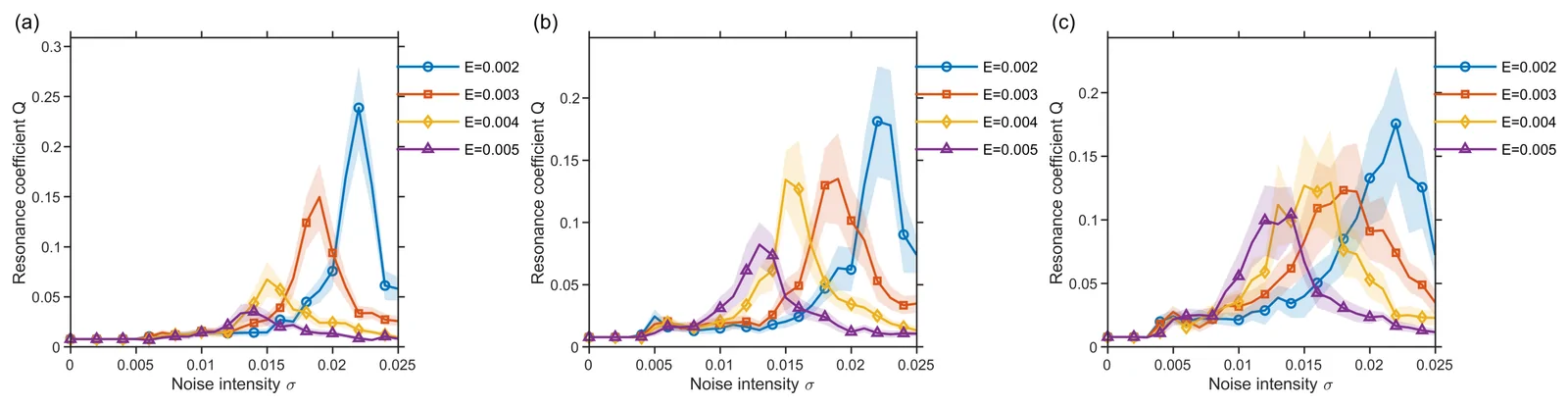
Effect of alternating electric-field amplitude on stochastic resonance under zero-delay conditions. The *Q*–σ relation is shown for E=0.002–0.005 at fixed field frequency f=0.05 and (**a**) $p_{t}=0.1$, (**b**) $p_{t}=0.3$, and (**c**) $p_{t}=0.5$. The periodic square-pulse input was applied to node 1. Lines and bands show means and two-sided 95% Student-*t* confidence intervals over 20 replicates.

**Figure 6:**
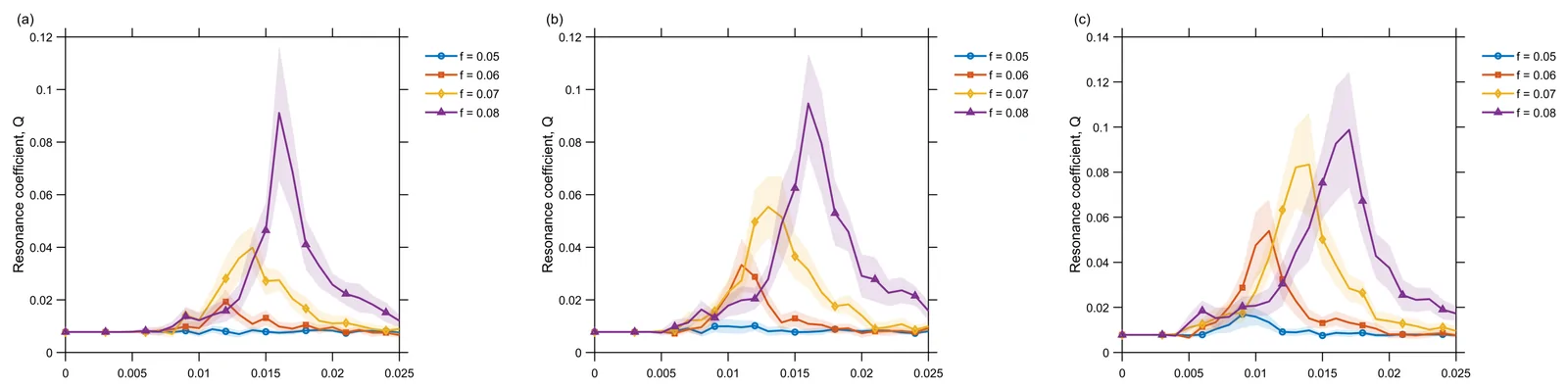
Effect of alternating electric-field frequency on stochastic resonance under zero-delay conditions. The *Q*–σ relation is shown for f=0.05–0.08 at fixed amplitude E=0.005 and (**a**) $p_{t}=0.1$, (**b**) $p_{t}=0.2$, and (**c**) $p_{t}=0.3$. The periodic square-pulse input was applied to node 1. Lines and bands show means and two-sided 95% Student-*t* confidence intervals over 20 simulation replicates.

The electric field acts on the fast neuronal dynamics, whereas the preceding scans varied synaptic or network properties. We therefore examined field amplitude and frequency under zero-delay conditions, tracking both the height of the SR peak and its location along the noise axis (Figure 5 and Figure 6). The two scans produced different response patterns.

At fixed f=0.05, the higher field amplitudes generally produced lower and earlier peaks along the noise axis (Figure 5). This pattern was most pronounced at $p_{t}=0.1$: increasing *E* from 0.002 to 0.005 reduced peak *Q* from 0.2387 to 0.0351, while the corresponding σ moved from 0.022 to 0.014. The curves at $p_{t}=0.3$ and 0.5 showed the same overall endpoint direction. At $p_{t}=0.5$, the intermediate amplitudes also produced a local rise between E=0.003 and 0.004.

Together, these curves show that field amplitude changed both how strongly the population followed the weak input and the noise intensity at which that response was greatest.

Changing field frequency at fixed E=0.005 produced the opposite endpoint pattern (Figure 6). From f=0.05 to 0.08, peak *Q* increased at all three examined compositions, while its associated noise intensity shifted upward. At $p_{t}=0.1$, peak *Q* rose from 0.00885 to 0.0911 and its σ from 0.011 to 0.016. The similar separation of the frequency curves across $p_{t}=0.2$ and 0.3 shows that this pattern was present throughout the tested composition range; the endpoint values are reported in Supplementary Table S1.

To assess the role of the frequency-dependent amplitude of the field-induced voltage term, we ran an additional zero-delay control with the amplitude of $V_{e}$ fixed at its value for $E_{0}=0.005$ and $f_{0}=0.05$, giving $V_{0}=E_{0}/\left(2\pi f_{0}\right)=0.0159155$. At each frequency, we set $A_{f}=V_{0}\left(2\pi f\right)$, preserving the original relation between $A_{f}$ and the amplitude of $I_{e}$; consequently, the latter increased from 0.005 to 0.008 over the tested range. With 20 replicates per $p_{t}$, peak *Q* increased from f=0.05 to 0.08 at all three compositions: 0.00870 to 0.01561 at $p_{t}=0.1$, 0.01076 to 0.02521 at $p_{t}=0.2$, and 0.01806 to 0.03502 at $p_{t}=0.3$. The corresponding peak-noise intensities changed from 0.012 to 0.015, 0.010 to 0.014, and 0.009 to 0.013. The increase was smaller than in the original fixed-*E* scan. The upward endpoint trend persisted with the amplitude of $V_{e}$ held constant; over this scan, the amplitude of $I_{e}$ increased from 0.005 to 0.008 with frequency. The control curves and peak summaries are provided in Supplementary Figure S1 and Table S4.

In the zero-delay network, amplitude and frequency therefore shifted the SR curves in different directions over their respective scanned intervals. The field-dependent response was assessed next in the presence of delayed synaptic input.

### 3.5. Effect of Transmission Delay on Noise-Dependent Stochastic Resonance

**Figure 7:**
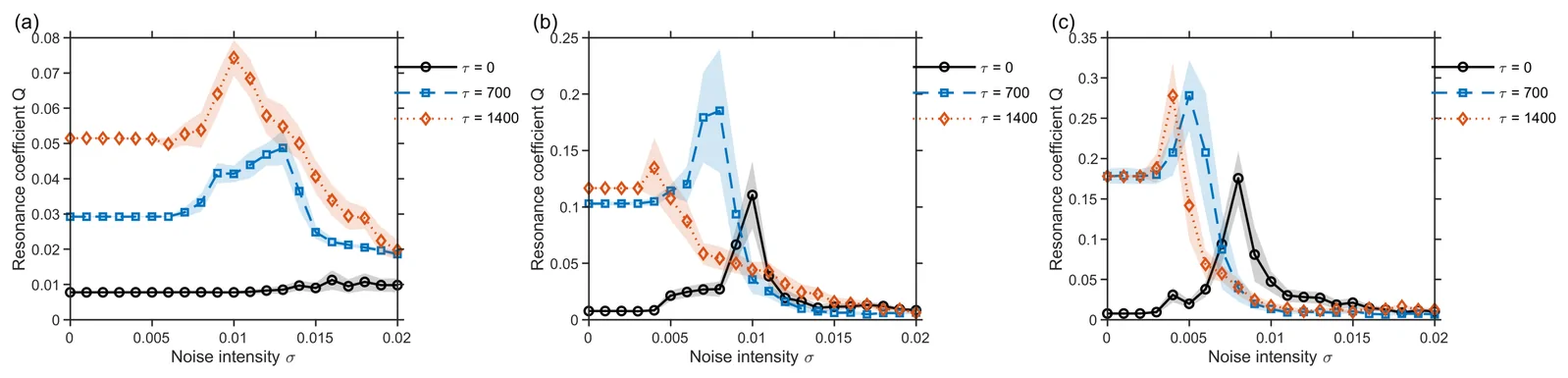
Stochastic-resonance curves at representative synaptic transmission delays. The *Q*–σ relations are shown for τ=0, 700, and 1400 at (**a**) $p_{t}=0$, (**b**) $p_{t}=0.5$, and (**c**) $p_{t}=1.0$, with $T_{s}=700$, E=0.005, and f=0.05. The periodic square-pulse input was applied to node 1. Lines and bands show means and two-sided 95% Student-*t* confidence intervals over 20 replicates.

Transmission delay changes the timing of synaptic input relative to the pulse train. We compared τ=0, 700, and 1400 at $T_{s}=700$ to determine how this timing affected the noise-dependent response in electrical-only, hybrid, and chemical-only networks (Figure 7). In the electrical-only network, both delayed curves rose above the zero-delay curve over much of the low-noise range. Its peak increased from 0.0113 at zero delay to 0.0743 at τ=1400, while the corresponding σ moved from 0.016 to 0.010.

The delayed curves were also higher in the hybrid and chemical-only networks, but the preferred delay changed with composition. At $p_{t}=0.5$, τ=700 gave the largest peak, 0.1852, whereas the electrical-only network reached its largest sampled peak at τ=1400. At $p_{t}=1$, the peaks at 700 and 1400 were nearly equal, approximately 58% above the zero-delay peak. The corresponding optimum noise intensities generally moved downward with delay. Hence, transmission delay enhanced the input-frequency response across these configurations while changing which delay was most favorable. The complete peak series is given in Supplementary Table S1.

### 3.6. Combined Effects of Transmission Delay and Electric-Field Parameters on the Response

**Figure 8:**
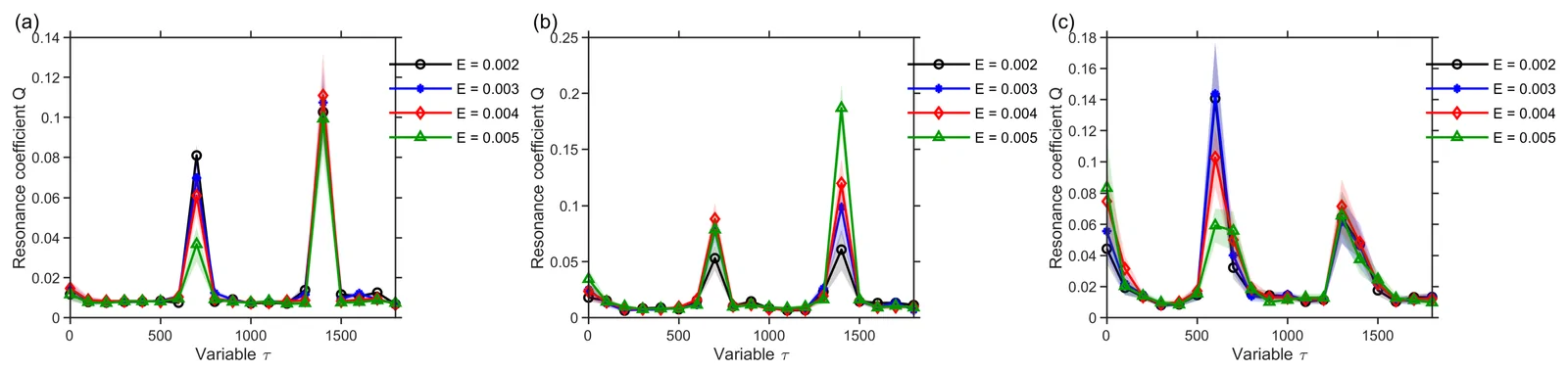
Joint dependence of the Fourier response amplitude on electric-field amplitude and transmission delay. *Q* is plotted against τ for E=0.002–0.005 at (**a**) $p_{t}=0.1$, (**b**) $p_{t}=0.5$, and (**c**) $p_{t}=1.0$, with f=0.07, σ=0.01, and $T_{s}=700$. The periodic square-pulse input was applied to node 1. Delays were sampled in 100-iteration increments. Lines and bands show means and two-sided 95% Student-*t* confidence intervals over 20 replicates.

**Figure 9:**
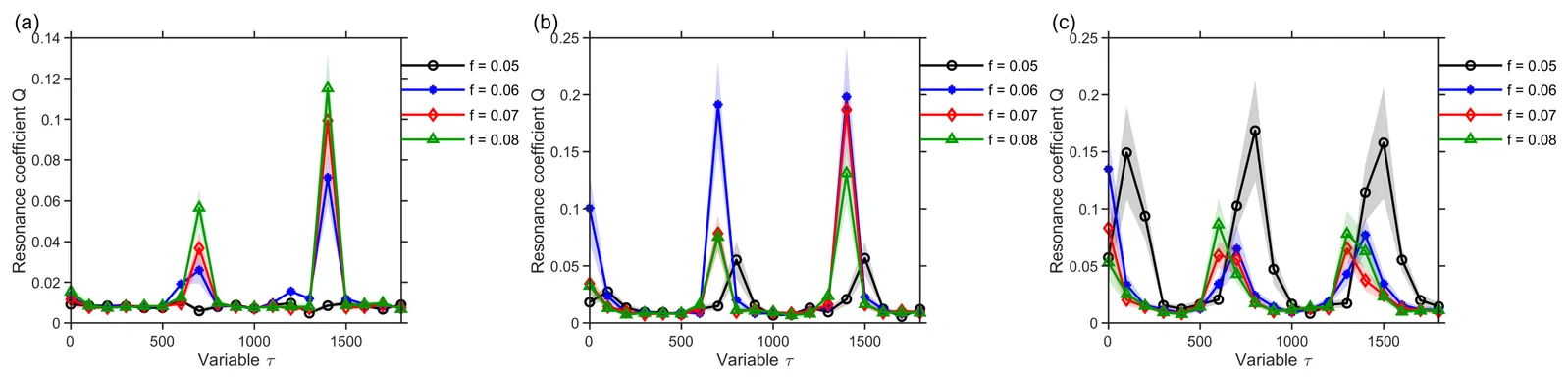
Joint dependence of the Fourier response amplitude on electric-field frequency and transmission delay. *Q* is plotted against τ for f=0.05–0.08 at (**a**) $p_{t}=0.1$, (**b**) $p_{t}=0.5$, and (**c**) $p_{t}=1.0$, with E=0.005, σ=0.01, and $T_{s}=700$. The periodic square-pulse input was applied to node 1. Delays were sampled in 100-iteration increments. Lines and bands show means and two-sided 95% Student-*t* confidence intervals over 20 replicates.

The preceding curves describe how delay changed the maximum reached during each noise-intensity scan. We next fixed σ=0.01 and followed *Q* across delay values so that the field settings could be compared under a common noise condition (Figure 8 and Figure 9). These delay scans reveal whether a field setting retains the same relative advantage as synaptic-input timing changes.

The amplitude scans displayed two prominent delay regions, near 700 and 1400 iterations, whose relative heights varied with $p_{t}$ (Figure 8). At $p_{t}=0.1$, the principal maximum remained at τ=1400 throughout E=0.002–0.005, whereas the response near τ=700 declined as amplitude increased. At $p_{t}=0.5$, increasing *E* had a different effect: *Q* at τ=1400 rose from 0.0608 to 0.1869. The same amplitude change therefore affected the two delayed response regions differently, and the direction of the long-delay change depended on composition.

The chemical-only network showed another ordering of the amplitude curves. At E=0.002 and 0.003, its largest values occurred at τ=600; at E=0.005, the largest value occurred at zero delay. Thus, varying amplitude changed the sampled delay of the strongest response as well as its height. The corresponding values are reported in Supplementary Table S1.

The frequency scans showed that the sampled field setting associated with the largest response changed across compositions (Figure 9). At $p_{t}=0.1$, *Q* at τ=1400 increased across the tested field settings as *f* rose from 0.05 to 0.08. At $p_{t}=0.5$, the largest sampled responses occurred at the joint setting f=0.06, reaching 0.1914 and 0.1982 at τ=700 and 1400. Because the field-induced voltage amplitude also varies with *f* when *E* is fixed, these values describe the response to the full field setting. Additional values appear in Supplementary Table S1.

At $p_{t}=1$, the setting f=0.05 produced the largest sampled delayed responses, with local maxima at τ=800 and 1500. Across the tested compositions, the field setting associated with the largest response differed under delayed conditions. Several prominent maxima lay near one or two input periods at $T_{s}=700$, motivating a direct comparison across input periods.

### 3.7. Effect of Input Period on Delay-Dependent Response Maxima

**Figure 10:**
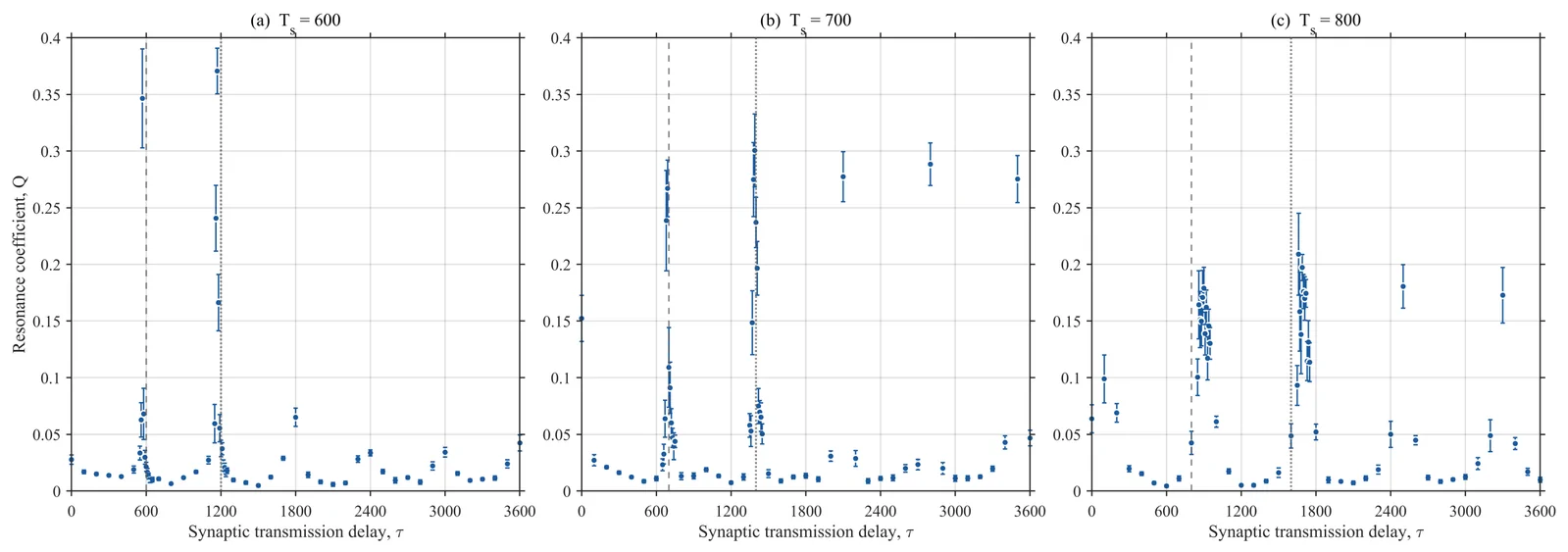
Input-period dependence of the delay-response relation. Panels (**a**–**c**) show the mean Fourier response amplitude *Q* against synaptic transmission delay τ for globally applied periodic inputs with $T_{s}=600$, 700, and 800, respectively; the other settings are $p_{t}=0.5$, E=0.005, f=0.07, and σ=0.01. Each symbol is the mean from 20 replicates, and its error bar is the two-sided 95% Student-*t* confidence interval. The coarse delay range is τ=0–3600 for all three periods, sampled at 100-iteration intervals. For $T_{s}=700,800$, the original 0–1800 scan and the added 1900–3600 scan use separate sets of 20 replicates; network and noise are shared across delays within each replicate and scan segment. Additional samples were taken every 10 iterations in intervals extending 50 iterations on either side of each of the two selected coarse-grid maxima in the original scans. Points are shown without connecting lines or smoothing. In each panel, the dashed and dotted vertical guides mark that panel’s $T_{s}$ and $2T_{s}$, respectively.

The delay maxima near $T_{s}$ and $2T_{s}$ at $T_{s}=700$ prompted a comparison across input periods. We extended the delay profiles for all three periods to τ=3600 (Figure 10). For $T_{s}=600$, the 100-iteration scan covered the full interval. For $T_{s}=700$ and 800, we retained the original τ=0–1800 data and added the interval τ=1900–3600 using 20 additional replicates. Within each scan segment, the network and noise realization were shared across delay values within a replicate; the original and added segments for $T_{s}=700$ and 800 used separate replicate sets. The settings were $p_{t}=0.5$, p=0.1, E=0.005, f=0.07, σ=0.01, and network-wide stimulation. Additional samples were taken every 10 iterations in intervals extending 50 iterations on either side of each of the two prominent coarse-grid maxima in the original scans. Figure 10 shows the full profiles; Supplementary Table S3 lists the endpoint values and local-refinement estimates.

The largest coarse-grid responses across the extended profiles occurred at τ=1800 (Q=0.0651), 2800 (Q=0.2884), and 2500 (Q=0.1805) for $T_{s}=600$, 700, and 800, respectively; the three highest peaks for each period are listed in Table 3. Endpoint responses and local-refinement estimates, including pointwise confidence intervals, are reported in Supplementary Table S3.

The refined maxima occurred at $0.95T_{s}$ and $1.95T_{s}$ for $T_{s}=600$, at $0.986T_{s}$ and $1.986T_{s}$ for $T_{s}=700$, and at $1.125T_{s}$ and $2.075T_{s}$ for $T_{s}=800$. In these conditions, the second maximum remained near $2T_{s}$, whereas the first maximum for $T_{s}=800$ occurred later than $T_{s}$.

**Figure 11:**
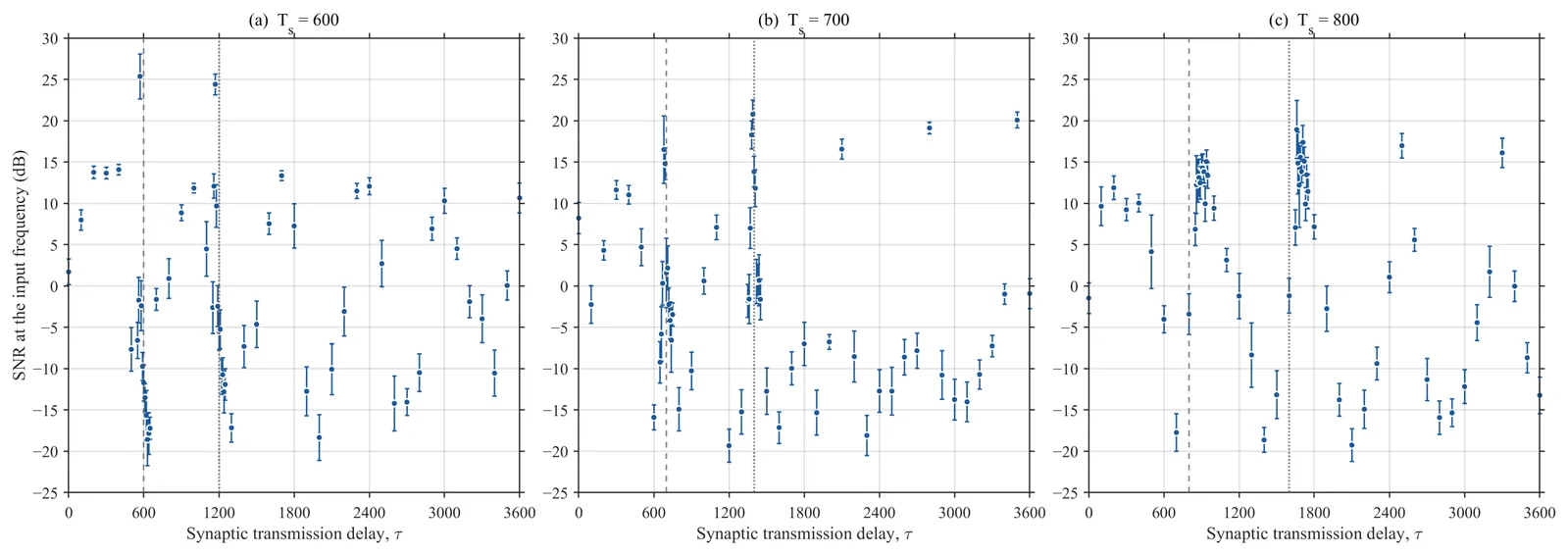
Input-period dependence of the delay-resolved spectral signal-to-noise ratio (SNR). Panels (**a**–**c**) show globally applied periodic inputs with $T_{s}=600$, 700, and 800, respectively; all other settings are N=200, K=3, p=0.1, $p_{t}=0.5$, $D_{e}=0.003$, $D_{c}=0.010$, E=0.005, f=0.07, and σ=0.01. Each symbol is the mean SNR from 20 replicates, and its error bar is the two-sided 95% Student-*t* confidence interval. The coarse delay range is τ=0–3600 for all three periods, sampled at 100-iteration intervals; for $T_{s}=700,800$, the original and added segments use separate sets of 20 replicates. The local peak intervals in the original scans were additionally sampled at 10-iteration intervals. Points are shown without connecting lines or smoothing. In each panel, the dashed and dotted vertical guides mark that panel’s $T_{s}$ and $2T_{s}$, respectively.

The delay-resolved profiles were also evaluated with spectral SNR to compare response amplitude with spectral contrast at the input frequency (Figure 11). Across the full sampled delay range, the largest SNR values occurred at τ=570 for $T_{s}=600$ (25.36 dB), 1390 for $T_{s}=700$ (20.77 dB), and 1660 for $T_{s}=800$ (18.93 dB). These locations coincide with one of the two largest refined *Q* peaks for each input period. This agreement indicates that, in these conditions, the strongest input-frequency component also had high contrast relative to neighboring spectral bins.

The two measures capture complementary response properties: *Q* quantifies the input-frequency component, whereas SNR quantifies its contrast with neighboring spectral bins. Their selected delays differed at $T_{s}=600$, while their largest responses fell within the same refined peak intervals.

## 4. Discussion

The central result is the composition-dependent response to delayed synaptic input and electric-field forcing. Earlier work separately examined hybrid synapses and rewiring [8,9,10,11], electric fields [16,17,18,19,20,21,22,23,24,25,26,29], and transmission delay [9,10,21,32,33,34,36,37]. Here, the field setting associated with the largest input-frequency response changed with $p_{t}$ and τ. This dependence connects synaptic composition and transmission timing to weak-signal responses within the present model.

Recent studies also support this parameter-dependent behavior. AC-induced electric fields have been shown to alter firing and phase locking as field amplitude and frequency vary [30], while hybrid electrical–chemical coupling can support different collective states as field frequency and coupling strength change [15]. Differences in neuron models, network structures, and response measures place the peak values from these studies in distinct modeling contexts.

Mechanistic explanations in this Discussion are hypotheses. The analyses quantify parameter-dependent response patterns, while the contributions of threshold-crossing dynamics, field forcing, recovery, and delayed propagation remain to be tested separately.

The parameters $p_{t}$, $D_{c}$, and $D_{e}$ represent distinct aspects of synaptic transmission. In this model, $p_{t}$ simultaneously changes synapse type, directionality, and threshold-dependent transmission. By comparison, $D_{c}$ and $D_{e}$ scale currents along existing chemical and electrical connections. The markedly larger response change across the investigated $D_{e}$ interval shows that electrical and chemical coupling strengths act as distinct control parameters in this network. This result is qualitatively consistent with simulations of a hybrid scale-free FHN network, in which electrical synapses were more effective than chemical synapses in enhancing the input–output correlation [7]. The differences in neuron models and network topologies define the scope of this qualitative comparison. Continuous voltage-difference coupling offers one possible explanation for the stronger $D_{e}$ dependence; its independent contribution remains to be assessed. The distinction is also consistent with simulations in which electrical coupling promoted ordered activity, whereas delayed inhibitory chemical coupling altered wave stability and synchronization [14]. That study complements the present excitatory-network results by illustrating differences between electrical and inhibitory chemical coupling mechanisms.

Field amplitude and the complete fixed-*E* field setting produced different zero-delay response patterns. As field amplitude increased, peak *Q* generally fell and its noise location shifted to lower intensities. Across the fixed-*E* frequency scan, peak *Q* rose with *f* over the tested range; in this scan, the amplitude of $V_{e}$ also changed with *f*. One possible, untested explanation is that field forcing and threshold crossings induced by the periodic input interact to shape these responses. Under delayed conditions, the relative responses to the sampled field settings varied with chemical-synapse probability.

The form of the electric-field terms determines the interpretation of the frequency dependence. Because the amplitude of $V_{e}$ in Equation (5) is $A/\omega_{e}$, varying *f* at fixed E≡A changes both the forcing frequency and the amplitude of the field-induced voltage term, whereas the amplitude of $I_{e}$ remains *A*. The original scan therefore characterizes the complete field formulation. In the additional zero-delay control, the amplitude of $V_{e}$ was fixed while $A_{f}$, and thus the amplitude of $I_{e}$, increased with frequency. The upward endpoint trend in peak *Q* persisted but was smaller than in the fixed-*E* scan. Across this control scan, the amplitude of $I_{e}$ increased with frequency. The separate delayed field-frequency scan retains fixed *E* and is interpreted as the response to the complete field formulation.

Watts–Strogatz rewiring simultaneously reduces the mean clustering coefficient and average shortest path length [4,5]. In the present scans, increasing *p* increased peak *Q* at $p_{t}=0.5$ while producing either a small decrease or a nonmonotonic response at lower $p_{t}$. Synaptic composition therefore determined the direction and form of the response to the combined topological changes produced by rewiring. Accordingly, the present results characterize the joint contribution of the concurrent changes in clustering coefficient and average shortest path length.

Multiple delay-dependent response maxima are consistent with previous reports of delay-induced resonance [9,10,21,32,33,34,36,37]. The locations of the two largest refined peaks changed with the input period: for $T_{s}=600$ and 700, they occurred near $T_{s}$ and $2T_{s}$, whereas for $T_{s}=800$, they occurred at 900 and 1660 ($1.125T_{s}$ and $2.075T_{s}$). Thus, the peak delays varied with the input period, and their period-normalized positions differed among the tested periods.

The recent literature likewise indicates that delay effects depend on noise, coupling architecture, and network structure. Bounded-noise simulations reported delay-dependent stochastic resonance under electromagnetic induction [38]; a feedforward network study found that signal propagation improved only at appropriate delays [42]; and an analysis of delayed multiplex networks showed that increasing interlayer delay generally suppressed the collective response [39]. The present results extend these observations by showing that the temporal structure of the input is another determinant of the delay-dependent response.

The Fourier response amplitude *Q* and spectral SNR characterize different properties of the response to the periodic input. *Q* measures the response component at the input fundamental frequency, whereas SNR measures its contrast with adjacent spectral bins. Across the extended profiles, the largest coarse-grid *Q* values occurred at τ=1800, 2800, and 2500 for $T_{s}=600$, 700, and 800, respectively, whereas the largest SNR values occurred at τ=570, 1390, and 1660. At $T_{s}=700$ and 800, the largest coarse-grid *Q* values fell in the added long-delay segments, while the SNR maxima remained within the original scan ranges. Thus, the delay associated with the largest Fourier component need not be the delay with the highest contrast against neighboring spectral bins.

Taken together, the results show that weak-signal enhancement varies with noise intensity and with the combination of electrical and chemical transmission, small-world connectivity, synaptic transmission timing, and external field forcing. Within the present model, rewiring effects vary with synaptic composition, the field setting associated with the largest response varies with composition and delay, and delay-dependent maxima shift with the input period.

In neural-stimulation or bio-inspired neuromorphic signal-processing systems, these parameter relationships may inform subsequent calibration and help define initial search ranges. With the target weak periodic input held fixed, stimulation amplitude, frequency, and synaptic transmission delay could be scanned according to the system’s adjustable range. Noise intensity could also be included when it can be controlled or characterized, while response amplitude at the input frequency or spectral SNR is recorded. In neuromorphic hardware, electrical and chemical coupling strengths could be implemented through programmable connection weights or branch gains; the chemical-synapse proportion could be represented by the configured ratio of connection types; and transmission delay could be set using programmable delay units or digital buffers. The noise-response intervals and delay intervals observed here may provide starting ranges for such scans, after which candidate configurations could be evaluated using system-specific performance criteria and operating constraints. Because this study is based on numerical simulations, these results provide model-level guidance for parameter search. Their applicability to neural-stimulation devices or neuromorphic hardware remains to be established through system calibration and experimental validation. Similar disturbance-compensation and state-estimation problems also arise in other engineering dynamical systems. For example, high-gain disturbance observers have been used for load-following control in pressurized-water reactors, and adaptive Kalman filters have been used to estimate unmeasured states in advanced passive reactor cores [43,44]. Although these studies address questions distinct from neuronal stochastic resonance, they illustrate, from a broader engineering-dynamical-systems perspective, the application of disturbance-handling and state-estimation methods across different systems.

These relationships characterize the Watts–Strogatz Rulkov network across the investigated parameter ranges. Electrical transmission is generally faster than chemical synaptic transmission [6], so the shared-delay assumption $\tau_{e}=\tau_{c}$ does not represent their distinct timescales; it provides a controlled comparison of delayed input. Testing separate electrical and chemical delays, together with inhibition, plasticity, and field amplitudes in physical units, would help assess whether these response patterns transfer to more biophysically detailed systems.

## 5. Conclusions

Intermediate noise maximized the input-frequency response across the examined noise-intensity scans. Synaptic composition shaped the response to rewiring and electric-field forcing, while delayed synaptic input changed the field-dependent response pattern.

The principal quantitative contrasts were a 9.7% increase in the Figure 1 peak mean between $p_{t}=0.3$ and 0.5 and a 67.6% increase at τ=700 relative to zero delay in the hybrid-coupled network. The confidence intervals for the Figure 1 peak means overlap. At fixed noise intensity, the field setting associated with the largest response depended on synaptic composition and delay. Several sampled delay maxima shifted as the input period changed.

Together, these results show that synaptic composition and synaptic transmission delay jointly shape the noise-assisted response to weak periodic signals in the modeled neuronal network. Their effects also depend on network topology and electric-field forcing. These relationships may help define candidate parameter ranges for input-frequency tuning in bio-inspired neural-signal-processing systems, but their applicability requires system-specific validation.

## Figures and Tables

**Table 1:** Shared model parameters and their definitions.

Parameter	Value	Definition
*N*	200	Number of Rulkov neurons in the network.
*K*	3	Number of initial ring-lattice neighbors on each side of a node.
α	1.95	Parameter in the fast-variable equation of the Rulkov map.
β,γ	0.001, 0.001	Parameters in the slow-variable equation of the Rulkov map.
$v_{i}$	0 or 1.5	Chemical-synapse reversal potential: 1.5 for nodes receiving at least one directed chemical synapse and 0 for the remaining nodes.
$\Theta_{s}$	−1	Center of the chemical-synapse sigmoid gating function.
λ	30	Steepness of the chemical-synapse sigmoid gating function.
$A_{s}$	0.0015	Amplitude of the periodic square-pulse input.
*w*	50	Width of each periodic square pulse.
$T_{s}$	700	Period of the square-pulse input.

**Table 2:** Mean clustering coefficient *C*, average shortest path length $L_{\mathrm{path}}$, and mean degree from 100 network realizations at each rewiring probability. Standard deviations quantify variability across realizations.

*p*	Mean *C*	SD *C*	Mean $L_{\mathrm{path}}$	SD $L_{\mathrm{path}}$	Mean Degree
0.0	0.6000	0.0000	17.0854	0.0000	6.0
0.1	0.4433	0.0193	4.3930	0.1546	6.0
0.3	0.2260	0.0189	3.4937	0.0413	6.0
0.5	0.0985	0.0115	3.2532	0.0198	6.0
0.7	0.0403	0.0066	3.1670	0.0102	6.0
1.0	0.0252	0.0050	3.1432	0.0092	6.0

**Table 3:** Three largest local maxima in the 100-iteration delay scan from τ=0 to 3600. Values are means and confidence-interval half-widths from 20 replicates; locally refined results are reported separately in Supplementary Table S3.

$T_{s}$	Rank	Peak Delay τ	Mean Response *Q*	95% CI Half-Width	Delay Ratio $\tau /T_{s}$
600	1	1800	0.0651	0.0080	3.000
600	2	1200	0.0375	0.0073	2.000
600	3	3000	0.0342	0.0043	5.000
700	1	2800	0.2884	0.0188	4.000
700	2	2100	0.2774	0.0221	3.000
700	3	3500	0.2753	0.0208	5.000
800	1	2500	0.1805	0.0192	3.125
800	2	900	0.1790	0.0185	1.125
800	3	1700	0.1760	0.0151	2.125

## Data Availability

The original contributions presented in the study are included in the article; further inquiries can be directed to the corresponding author.

## Supplementary Materials

The following supporting information can be downloaded at https://www.mdpi.com/article/10.3390/biomimetics11100733/s1: Table S1: Numerical values reported in the parameter scans; Table S2: Figure-specific scan conditions and replicate protocols; Figure S1: Zero-delay frequency control with fixed amplitude of the field-induced voltage term $V_{e}$; Table S3: Extended-scan endpoint responses and local delay maxima; Table S4: Peak response summaries for the original fixed-*E* frequency scan and the zero-delay fixed-$V_{e}$-amplitude control.

## References

[1] Benzi R., Sutera A., Vulpiani A. (1981). The mechanism of stochastic resonance. J. Phys. A Math. Gen..

[2] Gammaitoni L., Hänggi P., Jung P., Marchesoni F. (1998). Stochastic resonance. Rev. Mod. Phys..

[3] Douglass J.K., Wilkens L., Pantazelou E., Moss F. (1993). Noise enhancement of information transfer in crayfish mechanoreceptors by stochastic resonance. Nature.

[4] Watts D.J., Strogatz S.H. (1998). Collective dynamics of “small-world” networks. Nature.

[5] Bullmore E., Sporns O. (2009). Complex brain networks: Graph theoretical analysis of structural and functional systems. Nat. Rev. Neurosci..

[6] Connors B.W., Long M.A. (2004). Electrical synapses in the mammalian brain. Annu. Rev. Neurosci..

[7] Yilmaz E., Uzuntarla M., Ozer M., Perc M. (2013). Stochastic resonance in hybrid scale-free neuronal networks. Phys. A Stat. Mech. Its Appl..

[8] Wang J., Guo X., Yu H., Liu C., Deng B., Wei X., Chen Y. (2014). Stochastic resonance in small-world neuronal networks with hybrid electrical–chemical synapses. Chaos Solitons Fractals.

[9] Yu H., Guo X., Wang J. (2017). Stochastic resonance enhancement of small-world neural networks by hybrid synapses and time delay. Commun. Nonlinear Sci. Numer. Simul..

[10] Yu H., Wang J., Du J., Deng B., Wei X., Liu C. (2013). Effects of time delay and random rewiring on the stochastic resonance in excitable small-world neuronal networks. Phys. Rev. E.

[11] Li G., Sun X. (2017). Effects of hybrid synapses and partial time delay on stochastic resonance in a small-world neuronal network. Acta Phys. Sin..

[12] Liu C., Wang J., Yu H., Deng B., Wei X., Sun J., Chen Y. (2013). The effects of time delay on the synchronization transitions in a modular neuronal network with hybrid synapses. Chaos Solitons Fractals.

[13] Guo D., Wang Q., Perc M. (2012). Complex synchronous behavior in interneuronal networks with delayed inhibitory and fast electrical synapses. Phys. Rev. E.

[14] Xu Y., Li X. (2025). The pattern dynamics of interneuronal networks with inhibitory synaptic coupling. AIMS Math..

[15] Nguessap E.L.F., Roque A.C., Ferreira F.F. (2026). Dynamic effects of electric fields in a hybrid-coupled thermosensitive neuronal network. Phys. Rev. E.

[16] Herrmann C.S., Rach S., Neuling T., Strüber D. (2013). Transcranial alternating current stimulation: A review of the underlying mechanisms and modulation of cognitive processes. Front. Hum. Neurosci..

[17] Ali M.M., Sellers K.K., Fröhlich F. (2013). Transcranial alternating current stimulation modulates large-scale cortical network activity by network resonance. J. Neurosci..

[18] Wang X., Wang J., Deng B., Wei X., Li H.Y. (2013). The effects of induction electric field on sensitivity of firing rate in a single-compartment neuron model. Neurocomputing.

[19] Wang Y., Ma J., Xu Y., Wu F., Zhou P. (2017). The electrical activity of neurons subject to electromagnetic induction and Gaussian white noise. Int. J. Bifurc. Chaos.

[20] Du L., Cao Z., Lei Y., Deng Z. (2019). Electrical activities of neural systems exposed to sinusoidal induced electric field with random phase. Sci. China Technol. Sci..

[21] Yang X.L., Liu X.Q. (2019). How electromagnetic induction and coupled delay affect stochastic resonance in a modified neuronal network subject to phase noise. Int. J. Mod. Phys. B.

[22] Wang G., Yu D., Ding Q., Li T., Jia Y. (2021). Effects of electric field on multiple vibrational resonances in Hindmarsh–Rose neuronal systems. Chaos Solitons Fractals.

[23] Wu K., Li J. (2023). Effects of high–low-frequency electromagnetic radiation on vibrational resonance in FitzHugh–Nagumo neuronal systems. Eur. Phys. J. B.

[24] Wu K., Li J. (2024). Effects of electric field on vibrational resonance in Izhikevich neuronal systems. Chin. J. Phys..

[25] Zhang X., Min F., Dou Y., Xu Y. (2023). Bifurcation analysis of a modified FitzHugh–Nagumo neuron with electric field. Chaos Solitons Fractals.

[26] Wang X., Yu D., Li T., Jia Y. (2023). Logistic stochastic resonance in the Hodgkin–Huxley neuronal system under electromagnetic induction. Phys. A Stat. Mech. Its Appl..

[27] Yang H., Xu G., Wang H. (2022). Effects of magnetic fields on stochastic resonance in Hodgkin–Huxley neuronal network driven by Gaussian noise and non-Gaussian noise. Cogn. Neurodyn..

[28] Liu H., Lu L., Zhu Y., Wei Z., Yi M. (2022). Stochastic resonance: The response to envelope modulation signal for neural networks with different topologies. Phys. A Stat. Mech. Its Appl..

[29] Wu Y., Sun Z., Zhao N. (2025). Resonance dynamics in multilayer neural networks subjected to electromagnetic induction. Commun. Nonlinear Sci. Numer. Simul..

[30] Yuan C., Chen R., Li X., Zhao Y. (2025). Effects of AC induced electric fields on neuronal firing sensitivity and activity patterns. Front. Comput. Neurosci..

[31] Wang R., Jia Y., Li Y. (2025). Nonlinear cooperation between fast and slow dynamics underlying different subthreshold resonances in olive neurons model. Electron. Res. Arch..

[32] Yu D., Wang G., Ding Q., Li T., Jia Y. (2022). Effects of bounded noise and time delay on signal transmission in excitable neural networks. Chaos Solitons Fractals.

[33] Yu H., Li K., Guo X., Wang J., Deng B., Liu C. (2020). Firing rate oscillation and stochastic resonance in cortical networks with electrical–chemical synapses and time delay. IEEE Trans. Fuzzy Syst..

[34] Yu H., Guo X., Wang J., Li K., Deng B., Wei X., Liu C. (2020). Multiple stochastic resonances and oscillation transitions in cortical networks with time delay. IEEE Trans. Fuzzy Syst..

[35] Wu Y., Sun Z. (2024). Detecting stochastic multiresonance in neural networks via statistical complexity measure. Sci. Rep..

[36] He M., Li L., Jia W., Zhang J. (2025). Stochastic resonance in time-delayed bistable coupled network systems driven by Gaussian white noise. Chin. J. Phys..

[37] Yu D., Wu Y., Yang L., Zhao Y., Jia Y. (2023). Effect of topology on delay-induced multiple resonances in locally driven systems. Phys. A Stat. Mech. Its Appl..

[38] Shi R., Yang K., Hu X., Jin B., Gou Y., Wang C. (2025). Effects of time delay on stochastic resonance induced by sine–Wiener bounded noises in an improved Hindmarsh–Rose neuron model under electromagnetic induction. Eur. Phys. J. Spec. Top..

[39] Schlabritz A., Yamakou M.E. (2026). Interaction order controls stochastic-resonance enhancement and redistribution in delayed multiplex neural networks. arXiv.

[40] Rulkov N.F. (2001). Regularization of synchronized chaotic bursts. Phys. Rev. Lett..

[41] Rulkov N.F. (2002). Modeling of spiking-bursting neural behavior using two-dimensional map. Phys. Rev. E.

[42] Yang H., Zhang W., Bao J. (2026). Signal propagation of a feedforward neural network under electromagnetic stimulation. Chin. Phys. B.

[43] Hui J., Yuan J. (2022). High-gain disturbance observer-based double closed-loop sliding mode controller for the load following of a pressurized-water reactor. Prog. Nucl. Energy.

[44] Hui J. (2025). Adaptive Kalman filter-based real-time distribution estimation of unmeasured system states for an advanced passive reactor core under optimized mechanical shim control strategy. Expert Syst. Appl..

