# Supplementary material for: Stochastic Resonance in a Small-World Network of Rulkov Neurons with Electrical and Chemical Synapses Under an Alternating Electric Field and Synaptic Transmission Delay
Source: Biomimetics (Basel). 2026 Oct 14;11(10):733. doi: 10.3390/biomimetics11100733 (PMC13702100; doi:10.3390/biomimetics11100733)
Supplement: Supplementary file 1 [file biomimetics-11-00733-s001.zip › supplementary_peak_values.pdf]

Supplementary Table S1. Numerical values reported in the parameter scans

Values reproduce selected numerical results reported in the revised manuscript. A dash marks a peak location omitted from the source text. These selected values accompany the full curves in Figures 1–11 and summarize the peak values reported in the manuscript.

Figure	Condition	Parameter value	Response Q	Peak σ or delay τ
1	p_t	0.3 / 0.4 / 0.5	0.1089 / 0.1187 / 0.1194	$\sigma = 0.022$
2a	D_c	0.008 / 0.010 / 0.012	0.0448 / 0.0459 / 0.0485	$\sigma = 0.009$ / 0.010 / 0.009
2b	D_e	0.002 / 0.005 / 0.007	0.0352 / 0.0736 / 0.0937	$\sigma = 0.009$ / 0.010 / 0.011
3	$p_t = 0.1$, p	0.3 / 0.7	0.0286 / 0.0274	–
3	$p_t = 0.3$, p	0.3 / 0.5 / 0.7	0.0715 / 0.0780 / 0.0746	–
3	$p_t = 0.5$, p	0.3 / 0.5 / 0.7	0.1210 / 0.1367 / 0.1610	–
5	$p_t = 0.1$, E	0.002 / 0.005	0.2387 / 0.0351	$\sigma = 0.022$ / 0.014
5	$p_t = 0.3$, E	0.002 / 0.005	0.1813 / 0.0822	–
5	$p_t = 0.5$, E	0.002 / 0.005	0.1757 / 0.1041	–
5	$p_t = 0.5$, E	0.003 / 0.004	0.1233 / 0.1292	–
6	$p_t = 0.1$, f	0.05 / 0.08	0.00885 / 0.0911	$\sigma = 0.011$ / 0.016
6	$p_t = 0.2$, f	0.05 / 0.08	0.0101 / 0.0947	$\sigma = --$ / 0.016
6	$p_t = 0.3$, f	0.05 / 0.08	0.0169 / 0.0988	$\sigma = --$ / 0.017
7	$p_t = 0$, τ	0 / 700 / 1400	0.0113 / 0.0487 / 0.0743	$\sigma = 0.016$ / – / 0.010
7	$p_t = 0.5$, τ	0 / 700 / 1400	0.1105 / 0.1852 / 0.1348	$\sigma = 0.010$ / 0.008 / 0.004
7	$p_t = 1$, τ	0 / 700 / 1400	0.1756 / 0.2784 / 0.2781	$\sigma = 0.008$ / 0.005 / 0.004
8	$p_t = 0.1$, E	0.002–0.005	0.0994–0.1109	$\tau = 1400$
8	$p_t = 0.1$, E	0.002 / 0.005	0.0809 / 0.0368	$\tau = 700$
8	$p_t = 0.5$, E	0.002 / 0.005	0.0608 / 0.1869	$\tau = 1400$
8	$p_t = 1$, E	0.002 / 0.003	0.1407 / 0.1437	$\tau = 600$
8	$p_t = 1$, E	0.005	0.0834	$\tau = 0$
9	$p_t = 0.1$, f	0.06 / 0.07 / 0.08	0.0713 / 0.0994 / 0.1151	$\tau = 1400$
9	$p_t = 0.5$, f	0.06	0.1914 / 0.1982	$\tau = 700$ / 1400
9	$p_t = 0.5$, f	0.07 / 0.08	0.1869 / 0.1311	$\tau = 1400$
9	$p_t = 1$, f	0.05	0.1686 / 0.1578	$\tau = 800$ / 1500
