# Supplementary material for: Stochastic Resonance in a Small-World Network of Rulkov Neurons with Electrical and Chemical Synapses Under an Alternating Electric Field and Synaptic Transmission Delay
Source: Biomimetics (Basel). 2026 Oct 14;11(10):733. doi: 10.3390/biomimetics11100733 (PMC13702100; doi:10.3390/biomimetics11100733)
Supplement: Supplementary file 1 [file biomimetics-11-00733-s001.zip › supplementary_simulation_parameters.pdf]

**Common settings.** Unless varied below, $N = 200$, $K = 3$, $p = 0.1$, $\alpha = 1.95$, $\beta = \gamma = 0.001$, $D_e = 0.003$, and $D_c = 0.010$. Chemical gating and reversal potential are defined in Section 2, Equations (3)–(4). The pulse amplitude is $A_s = 0.0015$, its width is 50 iterations, and the default period is $T_s = 700$. Dynamic analyses use 20 replicates and two-sided 95% Student-$t$ confidence intervals ($df = 19$). Confidence intervals summarize total between-replicate variability under the listed resampling protocols. Figure 4 uses 100 networks per $p$ and reports mean ± SD for the topology metrics. Fourier-analysis windows, including the transient cutoff and the relation between record length and input period, are listed separately below.
**Fourier-analysis window settings by figure group.**

| Figure(s) | $T_s$ | $n_0$ | $n_1$ | $L$ | $L/T_s$ | Integer? |
|---|---|---|---|---|---|---|
| 1 | 700 | 20001 | 100000 | 80000 | 114.286 | No |
| 2–3, 5–9 | 700 | 5000 | 50000 | 45001 | 64.287 | No |
| 4 | – | – | – | – | – | N/A[a] |
| 10–11 | 600 | 5001 | 50000 | 45000 | 75 | Yes |
| 10–11 | 700 | 5201 | 50000 | 44800 | 64 | Yes |
| 10–11 | 800 | 5201 | 50000 | 44800 | 56 | Yes |

$n_0$ is the first iteration included after the transient; $n_0$ and $n_1$ are inclusive, so $L = n_1 - n_0 + 1$. "Integer?" indicates whether $L/T_s$ is an integer. [a]Figure 4 reports topology metrics only and has no Fourier-response window.

Table S2: Figure-specific scan conditions and replicate protocols.

| Figure | Scan and fixed conditions | Replicates and randomization |
|---|---|---|
| 1 | $p_t = 0.3, 0.4, 0.5$; $\sigma = 0 : 0.001 : 0.030$; local input to node 1; $T_s = 700$, $E = 0.005$, $f = 0.05$, $\tau = 0$. | Network and noise resampled for each $(p_t, \sigma)$ condition. |
| 2a, 2b | 2a: $D_c = 0.008, 0.010, 0.012$; 2b: $D_e = 0.002, 0.005, 0.007$; $p_t = 0.2$; $\sigma = 0 : 0.001 : 0.025$; network-wide input; $T_s = 700$, $E = 0.005$, $f = 0.05$, $\tau = 0$. | Network and noise resampled for each plotted condition. |
| 3 | $p = 0.3, 0.5, 0.7$; $p_t = 0.1, 0.3, 0.5$; $\sigma = 0 : 0.002 : 0.040$; local input to node 1; $T_s = 700$, $E = 0.005$, $f = 0.05$, $\tau = 0$. | Network, synapse assignment, and noise resampled for each parameter combination. |
| 4 | Topology only: $N = 200$, $K = 3$; $p = 0, 0.1, 0.3, 0.5, 0.7, 1.0$. | 100 networks per $p$; mean and SD of clustering coefficient, average path length, and mean degree. |
| 5 | $p_t = 0.1, 0.3, 0.5$; $E = 0.002, 0.003, 0.004, 0.005$; $\sigma = 0 : 0.001 : 0.025$; local input to node 1; $T_s = 700$, $f = 0.05$, $\tau = 0$. | Seed reset for each $p_t$. |
| 6 | $p_t = 0.1, 0.2, 0.3$; $f = 0.05, 0.06, 0.07, 0.08$; $\sigma = 0 : 0.001 : 0.025$; local input to node 1; $T_s = 700$, $E = 0.005$, $\tau = 0$. | One network per replicate, shared across frequency and noise conditions; Figure 6 intervals use the recalculated Student-$t$ values. |
| 7 | $p_t = 0, 0.5, 1.0$; $\sigma = 0 : 0.001 : 0.020$; $\tau = 0, 700, 1400$; $D_e = D_c = 0.01$; local pulse to node 1; $T_s = 700$, $E = 0.005$, $f = 0.05$. | One network per replicate; at each $\sigma$, the same noise realization is used across the three delays. |
| 8 | $p_t = 0.1, 0.5, 1.0$; $E = 0.002, 0.003, 0.004, 0.005$; $\tau = 0 : 100 : 1800$; local input to node 1; $T_s = 700$, $f = 0.07$, $\sigma = 0.01$. | One network per replicate, shared across the amplitude–delay scan. |
| 9 | $p_t = 0.1, 0.5, 1.0$; $f = 0.05, 0.06, 0.07, 0.08$; $\tau = 0 : 100 : 1800$; local input to node 1; $T_s = 700$, $E = 0.005$, $\sigma = 0.01$. | One network per replicate, shared across the frequency–delay scan. |
| 10/11 | Network-wide input; $T_s = 600, 700, 800$; $E = 0.005$, $f = 0.07$, $\sigma = 0.01$; $\tau = 0 : 100 : 3600$ for all periods, with 10-iteration refinement within $\pm 50$ iterations of the selected coarse peaks in the original scans. Figure 11 reports SNR from the input-frequency bin relative to ten adjacent bins on each side. | For $T_s = 600$, 20 replicates cover the full scan. For $T_s = 700, 800$, the original $\tau = 0$–1800 and added $\tau = 1900$–3600 segments use separate sets of 20 replicates; within each segment, one network and noise matrix per replicate are shared across all delay values. |

# Additional validation analyses

For the zero-delay fixed-$V_e$-amplitude control, the field-induced voltage term was written as $V_e(n) = V_0 \sin(2\pi f n)$, with $V_0 = E_0/(2\pi f_0) = 0.0159155$ set from the reference condition $E_0 = 0.005$, $f_0 = 0.05$. To preserve the original relation between the

common field parameter and the current term, the amplitude of $I_e(n) = A_f \cos(2\pi f n)$ was set to $A_f = V_0(2\pi f)$; it therefore ranges from 0.005 to 0.008 over the tested frequencies. The frequency grid was $f = 0.05, 0.06, 0.07, 0.08$, the noise grid was $\sigma = 0 : 0.001 : 0.025$, and 20 replicates were performed for each chemical-synapse probability $p_t = 0.1, 0.2, 0.3$. The remaining zero-delay simulation settings matched the Figure 6 scan.

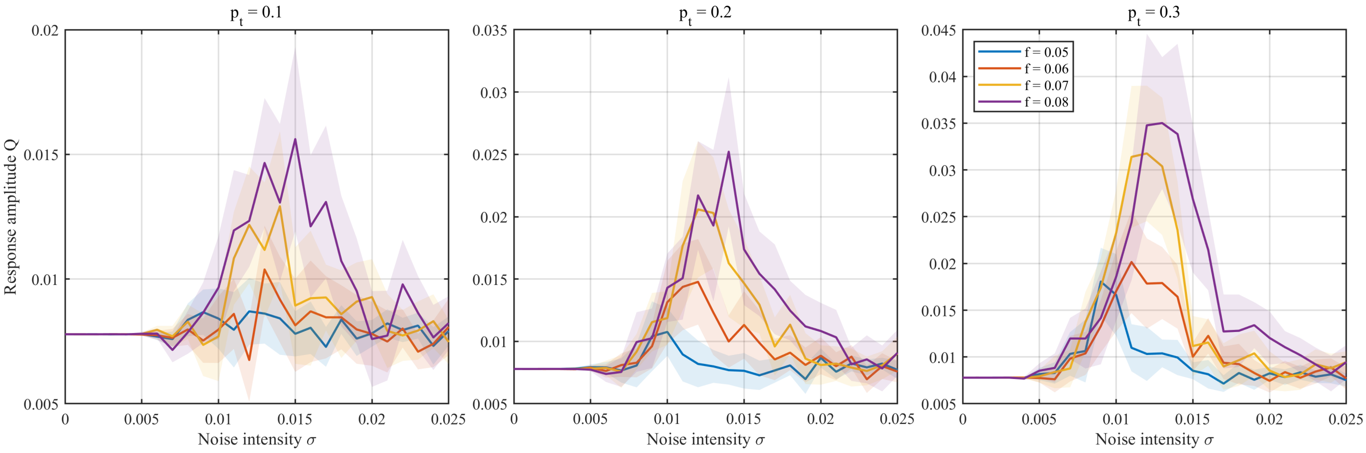


Figure S1: Zero-delay frequency control with fixed amplitude of the field-induced voltage term $V_e$. Mean response amplitude $Q$ is plotted against noise intensity $\sigma$ for $f = 0.05, 0.06, 0.07, 0.08$ and (left to right) $p_t = 0.1, 0.2, 0.3$; the periodic square-pulse input was applied to node 1. The amplitude of $V_e$ is fixed at $V_0 = 0.0159155$; the amplitude of $I_e$ follows $A_f = V_0(2\pi f)$. Bands denote two-sided 95% Student-$t$ confidence intervals over 20 replicates.

Table S3: Extended-scan endpoint responses and local delay maxima. The endpoint rows give the mean $Q$ at $\tau = 3600$ from the coarse scans; no CI half-width is listed for these rows. For refined peaks, the coarse center and refinement interval are listed; each interval extends 50 iterations on either side of a prominent maximum in the original scan. The reported 95% Student-$t$ CI half-width quantifies uncertainty in $Q$ at the selected delay grid point.

| $T_s$ | Estimate | Coarse center / interval | Delay $\tau$ | $\tau/T_s$ | Mean $Q$ | 95% CI half-width |
|---|---|---|---|---|---|---|
| 600 | Refined peak | 600 / 550–650 | 570 | 0.950 | 0.3465 | 0.0437 |
| 600 | Refined peak | 1200 / 1150–1250 | 1170 | 1.950 | 0.3706 | 0.0201 |
| 700 | Refined peak | 700 / 650–750 | 690 | 0.986 | 0.2670 | 0.0251 |
| 700 | Refined peak | 1400 / 1350–1450 | 1390 | 1.986 | 0.3005 | 0.0322 |
| 800 | Refined peak | 900 / 850–950 | 900 | 1.125 | 0.1790 | 0.0185 |
| 800 | Refined peak | 1700 / 1650–1750 | 1660 | 2.075 | 0.2090 | 0.0362 |
| 600 | Extended endpoint | – | 3600 | – | 0.0424 | – |
| 700 | Extended endpoint | – | 3600 | – | 0.0467 | – |
| 800 | Extended endpoint | – | 3600 | – | 0.0099 | – |

Table S4: Peak response summaries for the original fixed-$E$ frequency scan and the zero-delay fixed-$V_e$-amplitude control. Each peak is the largest mean $Q$ on the scanned $\sigma$ grid at that $(p_t, f)$; CI half-widths are two-sided 95% Student-$t$ intervals over 20 replicates.

| $p_t$ | $f$ | Fixed $E = 0.005$ | | | Fixed $V_e$ amplitude | | |
|---|---|---|---|---|---|---|---|
| | | Peak $\sigma$ | Peak $Q$ | CI half-width | Peak $\sigma$ | Peak $Q$ | CI half-width |
| 0.1 | 0.05 | 0.011 | 0.00885 | 0.00134 | 0.012 | 0.00870 | 0.00134 |
| 0.1 | 0.06 | 0.012 | 0.01932 | 0.00460 | 0.013 | 0.01038 | 0.00175 |
| 0.1 | 0.07 | 0.014 | 0.03989 | 0.00812 | 0.014 | 0.01292 | 0.00300 |
| 0.1 | 0.08 | 0.016 | 0.09110 | 0.02572 | 0.015 | 0.01561 | 0.00369 |
| 0.2 | 0.05 | 0.012 | 0.01015 | 0.00193 | 0.010 | 0.01076 | 0.00192 |
| 0.2 | 0.06 | 0.011 | 0.03326 | 0.01014 | 0.012 | 0.01477 | 0.00343 |
| 0.2 | 0.07 | 0.013 | 0.05540 | 0.01165 | 0.012 | 0.02059 | 0.00547 |
| 0.2 | 0.08 | 0.016 | 0.09469 | 0.01928 | 0.014 | 0.02521 | 0.00598 |
| 0.3 | 0.05 | 0.009 | 0.01695 | 0.00602 | 0.009 | 0.01806 | 0.00361 |
| 0.3 | 0.06 | 0.011 | 0.05390 | 0.01394 | 0.011 | 0.02015 | 0.00546 |
| 0.3 | 0.07 | 0.014 | 0.08341 | 0.02322 | 0.012 | 0.03177 | 0.00724 |
| 0.3 | 0.08 | 0.017 | 0.09878 | 0.02556 | 0.013 | 0.03502 | 0.00705 |
